# Complex Scrotal Reconstruction in Fournier′s Gangrene Using a Two‐Stage Delayed Bilateral Medial Thigh Skin Flap Technique: A Case Report

**DOI:** 10.1155/criu/5950371

**Published:** 2026-10-27

**Authors:** Souha Farhat, Samantha Cervantes Valadez, Alexa De la Fuente Hagopian, Tue A. Dinh

**Affiliations:** ^1^ Institute for Reconstructive Surgery Houston Methodist Hospital Weill Cornell Medicine Houston Texas USA cornell.edu

**Keywords:** case report, fasciitis, Fournier gangrene, necrotizing, reconstructive surgical procedures, scrotum, surgical flaps

## Abstract

Fournier′s gangrene is a rapidly progressive necrotizing soft tissue infection that can cause extensive scrotal loss and requires durable reconstruction with acceptable contour and function. A 54‐year‐old man presented 4 weeks after emergent perineoscrotal debridement with complete scrotal skin loss and exposed testes. Reconstruction was performed using a two‐stage approach. In Stage 1, each testis was placed into a medial thigh subcutaneous pocket beneath thin skin to encourage neovascularization. In Stage 2, delayed bilateral medial thigh skin flaps were elevated and advanced to the midline to create a neoscrotum. Minor donor‐site wound dehiscence was managed locally. A two‐stage delayed bilateral medial thigh skin flap can provide reliable, thin coverage and a natural neoscrotal contour after total scrotal loss.

## 1. Introduction

Fournier′s gangrene (FG) is a rare but rapidly progressive necrotizing fasciitis of the perineum, perianal region, and external genitalia, most commonly affecting men. Despite advances in care, mortality remains high, approaching 30% [1]. FG is typically a polymicrobial infection arising from a breach in gastrointestinal or genitourinary mucosa [2]. Early diagnosis, resuscitation, broad‐spectrum antibiotics, and aggressive debridement are crucial for survival. In men, extensive necrosis may result in major scrotal defects with exposed testes and spermatic cords, posing functional and psychological challenges.

Reconstruction after extensive debridement is complex and requires expertise in wound management to optimize functional and cosmetic outcomes. Techniques described include split‐thickness skin grafts and flap‐based reconstruction [3]. Among these, bilateral medial thigh flaps have been used successfully for scrotal and perineal reconstruction. However, flap bulkiness, sensory deficits, and occasional ischemia or necrosis have been reported [4].

In plastic surgery, delayed flap elevation is used to enhance vascularity and reduce ischemic complications, particularly in compromised wound beds. The delay phenomenon promotes neovascularization and improved blood flow, decreasing flap loss and increasing reliability [5]. A two‐stage delayed flap technique has been infrequently reported for scrotal reconstruction after FG. Given the high risk of flap failure in severely damaged tissue, a delayed approach may offer a survival advantage and improved functional and cosmetic outcomes.

We present the case of a 54‐year‐old African American male with complete scrotal tissue loss from FG, successfully managed with a novel two‐stage bilateral medial thigh flap reconstruction. This report outlines technical details, postoperative outcomes, and potential advantages of this approach.

## 2. Case Presentation

A 54‐year‐old male with hypertension presented 4 weeks after emergency debridement for FG. His initial surgery involved wide excision of necrotic perineoscrotal tissue with temporary coverage using an artificial skin graft (Figure 1). Despite antibiotics, persistent discomfort led to referral for definitive reconstruction.

**Figure 1:**
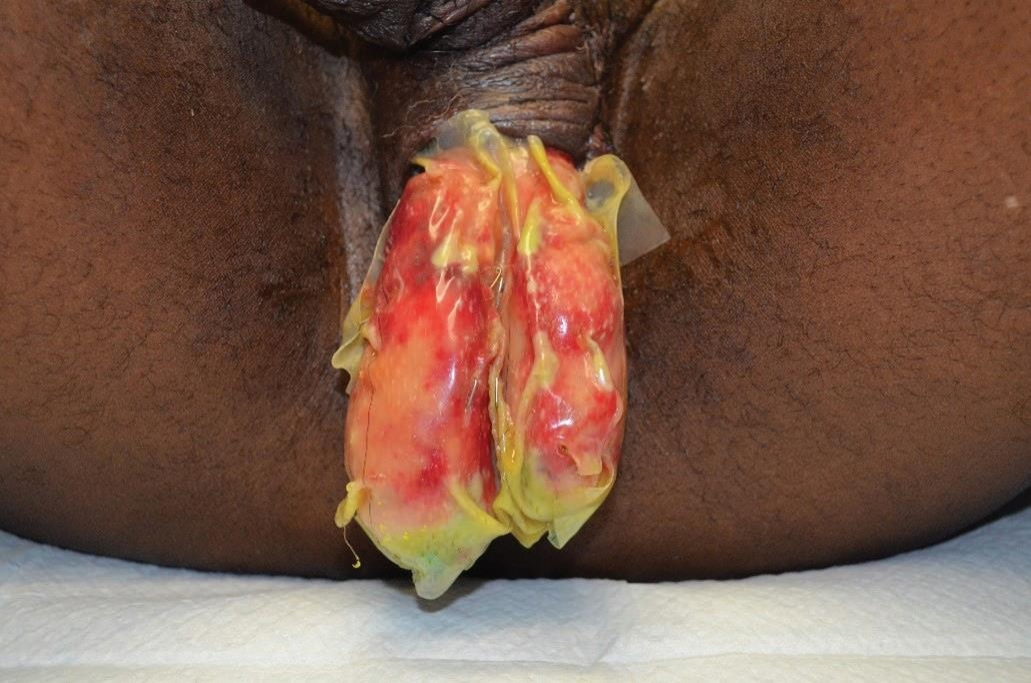
Exposed testes with complete absence of scrotal skin after separation of the artificial skin graft.

On examination, the patient had complete scrotal skin loss with exposed testes and spermatic cords. Both testes appeared viable with palpable pulses, no residual infection, and developing granulation tissue. Given his occupation as a long‐haul truck driver requiring prolonged sitting, and the need for durable, thin, protective coverage, flap‐based reconstruction was favored over skin grafting. Bilateral medial thigh tissue provided suitable donor sites.

After multidisciplinary discussion and separation of the artificial graft, a two‐stage bilateral medial thigh flap procedure was planned to optimize vascularity, reduce bulk, and replicate scrotal contour while preserving testicular function.

## 3. Treatment

### 3.1. Surgical Technique

#### 3.1.1. First Stage

With the patient supine, markings delineated the lateral surfaces of the testes and the medial thigh flaps, each measuring 11 × 9 cm (Figure 2). Subcutaneous pockets were created along the medial thighs extending from the testicular wound to the thigh. The skin was elevated to allow placement of each testis into its respective pocket, ensuring appropriate orientation beneath the medial thigh skin. Thin elevation of the skin over the bruised testes would allow formation of future scrotal sacs.

**Figure 2:**
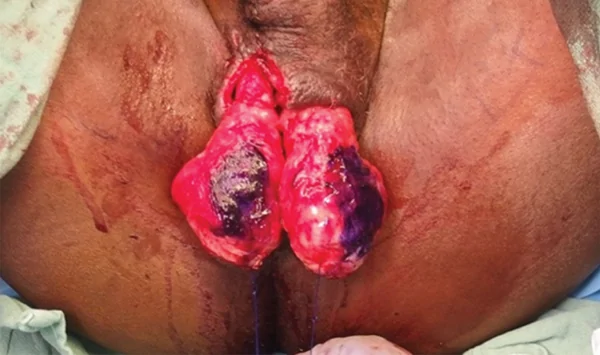
Preoperative marking of the lateral surfaces of both testes to ensure proper orientation beneath the medial thigh skin.

The medial thigh flaps were carefully sutured around the testes to promote revascularization. Two drains were placed in each pocket to prevent fluid accumulation. Remaining perineal tissue was advanced for closure, and a Foley catheter was maintained for urinary diversion (Figure 3A).

**Figure 3:**
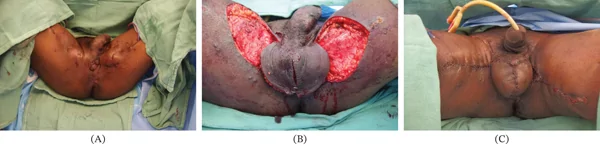
(A) Medial thigh pockets with subcutaneously positioned testes. (B) Elevated delayed medial thigh flaps, with exposed underlying tissue at the donor site, after medial rotation for scrotal reconstruction. (C) Medial thigh flaps rotated and sutured at midline for posterior and lateral scrotum reconstruction, with a Foley catheter in place.

#### 3.1.2. Second Stage (3 Weeks Later)

The second stage involved flap elevation and scrotal reconstruction by advancing both delayed skin flaps to the midline. Flaps were elevated from distal to proximal and advanced medially to create a neoscrotum (Figure 3B). Doppler ultrasound confirmed testicular blood flow.

The flaps were approximated in the midline and sutured to the testes with layered closure using 2‐0 PDS for dermal layers and 3‐0 chromic for the skin. A slim residual flap was used to cover the anterior scrotal wound and penile base. Donor sites were closed with tissue advancement, and drains were placed as needed (Figure 3C). No intraoperative complications occurred.

## 4. Outcome and Follow‐up

After the second stage, the patient was monitored in the hospital and discharged on postoperative Day 2 with oral antibiotics and pain control. Minor complications included superficial dehiscence at the right thigh donor site, managed with local care and a rotational advancement flap, and transient right lower extremity edema, which resolved conservatively. The scrotal flaps remained viable with continued improvement at the donor site. Figure 4 shows the healed patient at 9 weeks postoperatively.

**Figure 4:**
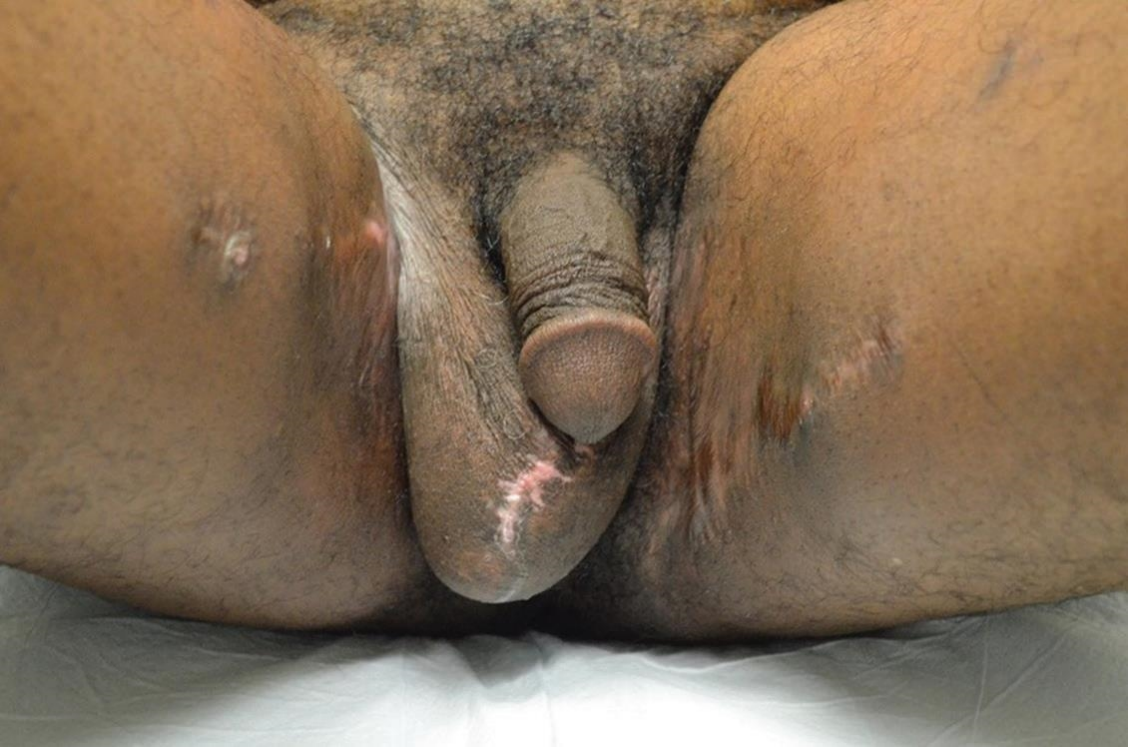
Nine weeks postoperative status showing appropriate healing of all incisions and donor sites.

At 1 year, the neoscrotum demonstrated good functional and sensory recovery. The patient reported no pain, no sexual dysfunction, and full return to normal activities and occupation.

## 5. Discussion

FG is one of the most challenging reconstructive scenarios because lifesaving debridement often results in major tissue loss with exposed testes and spermatic cords. Reconstruction must ensure durable coverage while restoring scrotal function, including thermoregulation, testicular protection, and psychosocial comfort. Technique selection is critical, as inadequate reconstruction can compromise physiological and psychological outcomes.

Options include skin grafts, local fasciocutaneous flaps, and free flaps. Skin grafts in the perineum are often unsatisfactory because of contamination, moisture, and pressure, leading to maceration and breakdown [3]. Many flaps have been described, but bulkiness remains a major drawback [6]. Increased tissue thickness raises testicular temperature, risking impaired spermatogenesis. The scrotum must maintain testicular temperature 2°C–4°C below core temperature for normal function [7]. Culp and Huffman′s 1956 findings demonstrated that placing testes directly beneath thigh skin, rather than under subcutaneous fat, preserved spermatogenesis [8].

Although most techniques are performed as one stage, our two‐stage approach may offer several advantages. Bilateral medial thigh skin flaps were selected for their reliable blood supply based on testicular vessels, durability, and expandability—important for a patient with prolonged sitting demands. Although Hallock previously described medial thigh fasciocutaneous flaps for scrotal reconstruction, a delayed medial thigh skin technique was chosen here for superior functional and structural outcomes [9].

Functionally, thin skin allows the testes to hang naturally, avoiding the added weight of thicker flaps and better replicating normal scrotal anatomy. This mobility supports optimal temperature regulation and reproductive physiology. Cosmetically, thin skin provides a more natural contour.

Regarding sensation, scrotal innervation varies but generally derives from lumbar and sacral plexus branches. The anterior scrotum is supplied by the genitofemoral nerve, whereas posterior sensation comes from perineal branches of the pudendal nerve and the posterior femoral cutaneous nerve. In this case, the flaps demonstrated good sensory recovery, likely because of shared segmental innervation between medial thigh skin and the scrotum, primarily via ilioinguinal and genitofemoral nerves, facilitating spontaneous reinnervation [10].

Complications in FG reconstruction require a multidisciplinary approach involving urology, plastic surgery, and, when needed, critical care. Early plastic surgery involvement is essential for planning reconstruction. Although complex reconstruction may not fully restore normal anatomy, this patient achieved good functional recovery with preserved testicular function despite minor delayed wound‐healing issues that resolved appropriately.

## 6. Conclusion

This case demonstrates that a two‐stage delayed bilateral medial thigh flap can successfully restore scrotal form and function after total tissue loss from FG. The staged approach enhances flap reliability, minimizes bulk, and closely replicates natural scrotal anatomy, allowing for potentially preserved testicular thermoregulation and function. Although donor‐site morbidity and the need for multiple operations are limitations, this technique represents a valuable reconstructive option for patients with extensive FG defects. Early multidisciplinary planning remains essential for optimal outcomes.

## Author Contributions

**Souha Farhat:** conceptualization, data curation, investigation, writing – original draft, writing – review and editing. **Tue A. Dinh:** conceptualization, methodology, supervision, writing – review and editing. **Samantha Cervantes Valadez:** data curation, investigation, writing – review and editing. **Alexa De la Fuente Hagopian:** data curation, investigation, writing – review and editing.

## Funding

No funding was received for this manuscript.

## Disclosure

All content was reviewed, revised, and approved by the authors, who take full responsibility for the final manuscript.

## Ethics Statement

This report describes the clinical course and surgical management of a single patient. In accordance with institutional policy, IRB approval was not required for publication of a single‐patient case report. Written informed consent for publication of clinical details and images was obtained from the patient.

## Consent

Written informed consent for publication of all clinical details and associated images was obtained from the patient in accordance with institutional requirements and applicable data protection and privacy regulations, including HIPAA.

## Conflicts of Interest

The authors declare no conflict of interest.

## Supporting information


**Supporting Information** Additional supporting information can be found online in the Supporting Information section. File S1: CARE checklist of information to include when writing a case report.

## Data Availability

The data that support the findings of this study are available on request from the corresponding author. The data are not publicly available due to privacy or ethical restrictions.
